# Impact of Pregnancy on Self-Efficacy and Personal Competence in the Context of Risk of Depression, Mental Health Status, and Satisfaction with Life

**DOI:** 10.3390/jcm13020533

**Published:** 2024-01-17

**Authors:** Agnieszka Kułak-Bejda, Ilknur Avci Aydin, Dilek Çelik Eren, Lambrini Kourkouta, Areti Tsaloglidou, Konstantinos Koukourikos, Andriej Szpakow, Natallia Khvoryk, Liudmila Hutsikava, Napoleon Waszkiewicz

**Affiliations:** 1 Department of Psychiatry, Medical University of Bialystok, 15269 Bialystok, Poland; napoleonwas@yahoo.com; 2 Nursing, School of Health, Ondokuz Mayis University, Samsun 55139, Turkey; ilknursezera@hotmail.com (I.A.A.); dilek.celik@omu.edu.tr (D.Ç.E.); 3 Department of Nursing, International Hellenic University, 57400 Sindos, Greece; laku1964@yahoo.gr (L.K.); aretitsa2010@hotmail.com (A.T.); kokaea@yahoo.gr (K.K.); 4 Department of Integrated Medical Care, Medical University of Bialystok, 15269 Bialystok, Poland; andrei.shpakou@umb.edu.pl; 5 Department of Obstetrics and Gynecology, Grodno State Medical University, 230009 Grodno, Belarus; kafedra2.bsmp@mail.ru (N.K.); klam4@mail.ru (L.H.)

**Keywords:** postpartum, pregnancy, depression, life satisfaction, mental health

## Abstract

For many women, pregnancy and childbirth are often accompanied by strong emotions related to fear, stress, and anxiety about the health of the woman and her child. This study aimed to assess the effect of pregnancy on the risk of depression, mental health status, and satisfaction with life in women in Poland, Greece, Turkey, Belarus, and Russia. Material and methods: A cross-sectional comparative study was conducted among 2017 women surveyed, including 584 pregnant women, 528 postpartum women, and 906 women who had never been pregnant (the comparative group) from Poland, Greece, Turkey, Belarus, and Russia. The sample selection was purposive. Surveys were collected between November 2021 and December 2022. The study used the Beck Depression Inventory (BDI) Scale, the Satisfaction with Life Scale (SWLS), the Edinburgh Postpartum Depression Scale (EPDS), the GHQ-28 (General Health Questionnaire), the Schwarzer Generalized Self-Efficacy Scale (GSES), and the KompOs Personal Competence Scale. Results: A significantly lower risk of depression was observed in postpartum women in Poland and amongst pregnant women in Turkey. Pregnant women in Turkey (28.9 points) presented the highest satisfaction with life, while the lowest satisfaction was found amongst pregnant women in Poland and Greece (25.2 and 25.1 points, respectively). In Poland and Belarus, a higher risk of depression was noticed in women who had undergone an artificial abortion. In Turkey, a positive correlation was found in pregnant women concerning the number of children they had with a measure of depression and a negative correlation with life satisfaction. In Greece, non-pregnant women showed no correlation between mental status and scores on the GSES. Scores on satisfaction with SWLS were positively correlated with a sense of power, and the strength of the correlation was similar to results on the BDI and GHQ measures. Postpartum depression, according to the EPDS, was also the most severe in Turkish women. Conclusions: The highest risk of depression was shown in the control group and amongst pregnant and postpartum women in Turkey and Greece, and the lowest such risk was in Poland. Pregnant and postpartum women showed by far the highest satisfaction with life in Turkey and the lowest in women from Greece. The risk of depression, the level of satisfaction with life, and the mental health of pregnant women were not influenced by the type of last delivery. However, the duration of the last delivery influenced the group from Belarus, and having children affected the mental health of women in the group from Turkey.

## 1. Introduction

A demographic crisis has been escalating in many countries worldwide for many years. Fertility rates, despite some governments’ pro-family policies, have been decreasing steadily. In 2021, the number of births in South Korea, with a population of 57 million, fell by more than 4% to a record low of 261,000. Among the 38 countries of the world grouped in the OECD, where the fertility rate averages 1.59 (figures for 2020), Poland averages only 1.38 children per woman. The highest fertility rate in the OECD is in Israel, with a rate of 2.9 children per woman.

Generational replacement, which prevents a country’s population from shrinking, is statistically ensured by a fertility rate close to 2.1. In the OECD, apart from Israel, this level is exceeded only in South Africa, Indonesia, Saudi Arabia, Argentina, Peru, and India. Russia’s fertility rate is 1.83, and China’s is 1.70. In the European Union, higher fertility rates than Poland’s 1.38 are found in France (1.79), Latvia (1.74), Czechia (1.71), Lithuania (1.69), and Sweden (1.67). In contrast, Italy (1.24) and Greece (1.28) rank lower. The country with the lowest fertility rate in the world is South Korea, with a rate of 0.9 children per woman [1].

**Figure 1:**
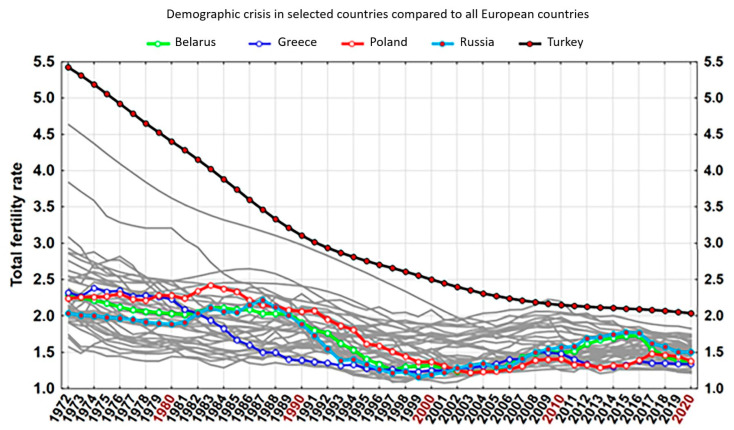
Fertility rates in the countries included in the analysis over the last half-century.

Figure 1 shows fertility rates in the countries included in the analysis over the past half-century. The data are from World Bank databases [2].

Pregnancy is a unique period in every woman’s life, as she lives in the hope that her child will be born perfectly healthy and on schedule. Consequently, any fertility issues are perceived as a violent shattering of previous expectations about the course of the pregnancy, as well as a failure to fulfil the parental role. For many women, both pregnancy and subsequent motherhood are natural stages of development. Consequently, for most of them, fertility issues are a source of strong emotional responses and intensified stress.

Pregnancy and childbirth represent a turning point in the lives of many women, especially primiparous women. It is often accompanied by strong emotions of fear, stress, and anxiety about their own and their baby’s health. Many women fear the pain, suffering, and complications of childbirth [3]. According to research, fear of childbirth can be described as a manifestation of prenatal stress, an anxiety disorder, or fear of a phobic nature manifesting itself in the form of night terrors, physical complaints, and difficulty concentrating on work and household activities [4]. Prenatal anxiety may be associated with pregnancy and childbirth itself, being a parent, or a personal tendency to react with anxiety [5]. Anxiety during pregnancy, on the other hand, is linked to the state of pregnancy, childbirth, and hospitalisation [6].

Depression, a mood disorder that causes a persistent feeling of sadness and loss of interest, is the most common mood disorder in the general population. The condition occurs twice as often in women as in men, and the initial onset of depression peaks during a woman’s reproductive years [7]. Life satisfaction is a subjective, cognitive evaluation of an individual’s life as a whole based on the fit between personal goals and achievements [8]. Satisfaction with life is one of the main dimensions of mental health. Satisfaction with life may play a role in the development of depressive symptoms.

To the best of our knowledge, similar studies from Belarus are unavailable. A recent study from Russia [9] assessed perinatal depressive disorders amongst 343 pregnant women. In this study, 36.4% of participants suffered from prenatal depression, and 34.3% of participants had postnatal depression. This Russian study identified that physical well-being during pregnancy and marital satisfaction during pregnancy significantly predicted prenatal depression. Birth satisfaction, physical well-being at two months after delivery, and marital satisfaction during pregnancy and after delivery significantly predicted postnatal depression. Moreover, this was the first study of perinatal depressive disorders in the Russian sample. In a Greek study [10], depressive symptoms were evaluated among pregnant women hospitalized during a high-risk pregnancy. Pregnant women admitted at >24 and <36 gestational weeks were eligible to participate in the study. The prevalence of antenatal depression was 24.3%. In the multivariate analysis, antenatal depression was significantly correlated with thoughts of abortion. In a Turkish study [11], the prevalence of postpartum depression and related risk factors were evaluated in a systematic review and meta-analysis. It was found that the prevalence of postpartum depression was 24%. The most commonly reported risk factors of postpartum depression were depression prior to pregnancy, unwanted pregnancy, low socioeconomic level, bad marital problems with a spouse, and being a housewife. In a Polish study from 2023 [12], the risk and severity of depression in pregnant and postpartum women were evaluated. The severity of depressive disorders was assessed using the Edinburgh Postnatal Depression Scale, Depression Inventory-Second Edition, and Hospital Anxiety and Depression Scale. A higher rate of depression in women in the third trimester of pregnancy was reported compared to women in the first week after delivery.

### Aim of the Study

The study aimed to assess the effect of pregnancy on the risk of depression, mental health status, and satisfaction with life in women in Poland, Greece, Turkey, Belarus, and Russia.

For the research hypotheses, we compared pregnant women and postpartum women to non-pregnant and nulliparous women (the comparative group). These were the hypotheses:

1. Women’s predisposition to depression, including postpartum depression differs between the countries surveyed. 2. Women’s life satisfaction differs between the countries surveyed. 3. Women’s experience of the severity of difficulties varies by nationality. 4. Women’s sense of self-efficacy differs between countries.

## 2. Material and Methods

### 2.1. Research Design

A cross-sectional study was carried out amongst the 2017 women surveyed, including pregnant women and nulliparous women (as the comparative group) from Poland, Greece, Turkey, Belarus, and Russia. The sample selection was purposive.

### 2.2. Participants

A total of 2017 women were surveyed, including 584 pregnant women, 528 postpartum women, and 906 nulliparous women (the comparative group) from Poland, Greece, Turkey, Belarus, and Russia.

The pregnant women were recruited from Obstetrics Departments, the postpartum women were from GP clinics, and the control group included female students and workers at universities in the studied countries. Written informed consent for participation in the study was obtained from all participants. The participation was voluntary and anonymous.

Inclusion criteria: pregnant and postpartum women were included in the study if they were over 18 years of age, willing to give informed consent, and could communicate in the mother language of the country where they were contacted. Exclusion criteria: women were excluded if they were below 18 years of age, did not give informed consent, or could not communicate in the mother language of the country where they were contacted.

### 2.3. Measures

The study used the following questionnaires:

The Beck Depression Inventory to assess the presence and severity of depressive symptoms [13]—all groups. It is used to self-assess the presence and severity of depression symptoms. The scale consists of 21 points rated according to the intensity of symptoms, from 0 to 3. For each point, the respondent should select one answer that, in his opinion, best describes his condition in the indicated period (before the doctor asks the patient to complete the scale, he should specify what period the answers should refer to; a month, a week, or the last 24 h). Obtaining from 0 to 11 points indicates no depression, 12–26 points indicates a mild depressive episode, 27–49 points indicates a moderate depressive episode, and 50–63 points indicates a major depressive episode. The Cronbach’s alpha coefficient was 0.93 and 0.95 for the entire scale.

The Satisfaction With Life Scale—SWLS [14]. The scale contains five statements. The respondents assessed the extent to which each of them related to his/her life so far, where 1—I completely disagree, 2—I do not agree, 3—I rather disagree, 4—I neither agree nor disagree, 5—I rather agree, 6—I agree, and 7—I completely agree. The obtained ratings were added up, and the overall result indicated satisfaction with one’s own life. The range of results could be from 5 to 35 points, and the higher the result, the greater the sense of satisfaction with life, where a score of 5–9 points Indicates a person dissatisfied with their life, 10–14 points—a person very dissatisfied with their life, 15–19 points—a person rather dissatisfied with their life, 20 points—a person neither satisfied nor dissatisfied with their life, 21–25 points—a person rather satisfied with their life, 26–30 points—a person very satisfied with their life, 31–35 points—a person satisfied with their life. The reliability index (Cronbach’s alpha) of the SWLS, established in a study of 371 adults, is 0.81. The scale constancy index, determined by testing a group of 30 people twice with an interval of six weeks, was 0.86.

The Edinburgh Postnatal Depression Scale—EPDS [14,15]. The Edinburgh Postnatal Depression Scale was developed to assist health professionals in detecting mothers suffering from postpartum depression, a distressing disorder more prolonged than the “blues” (which can occur in the first week after delivery). The scale consists of 10 questions, each with four ready-made answers. The woman is asked to read each statement and choose the one that best reflects how she has felt over the past seven days. The maximum number of points is 30. Mothers scoring above 12 or 13 are likely to be suffering from depression and should seek medical attention, sensitivity, and specificity at the level of 84.2–93.9% and 75.2–76.7%, and Cronbach’s alpha—0.87–0.88%.

The General Health Questionnaire GHQ-28 [16,17]. The scale is used to assess the state of mental health. It allows for the identification of people whose mental state has undergone a temporary or long-term breakdown due to experienced difficulties, problems, or mental illness and those at significant risk of mental health disorders. The questionnaire consists of 28 questions measuring four symptom areas: somatic disorders (A), anxiety (B), functional disorders (C), and depression (D). Theoretical scores for the scales range from 7 to 28 points; the higher the score, the greater the difficulties experienced. Cronbach’s alpha coefficients are 0.955, 0.956, 0.945, and 0.926, respectively. The GHQ-28 scale examines four dimensions of mental state: A—somatic symptoms (Cronbach’s alpha for the study group = 0.876), B—anxiety and insomnia (alpha = 0.916), C—social functioning disorders (alpha = 0.933), D—symptoms of depression (alpha = 0.941), which together give an overall score.

The Generalised Self-Efficacy Scale (GSES) by Schwarzer et al. (GSES) [14]. The GSES comprises 10 statements and measures the intensity of one’s general opinion on their efficacy in dealing with difficult situations and obstacles. The total score presents a general self-efficacy indicator, ranging from 10 to 40 points. High scores represent high self-efficacy. The Cronbach’s α of the scale is high—0.85.

Personal Competence Scale—Kompos [12]. KompOs measures generalized self-efficacy. It also allows to obtain results in two subscales: the disposition of the force necessary to initiate the action and the perseverance necessary to continue the action. The internal consistency of the KompOs, as assessed using Cronbach’s alpha, is 0.72 for the entire Scale, 0.74 for the strength subscale, and 0.62 for perseverance.

### 2.4. Procedures

A total of 2017 women were surveyed. Surveys were collected between November 2021 and December 2022. The actual number of responses is reported in the individual analyses. The questionnaires were given to the participants by the authors of the study. All completed questionnaires from Greece, Turkey, Belarus, and Russia were sent to Poland. A statistician analysed the data from the questionnaires.

Participation in the study was voluntary. All participants could withdraw from or leave the study at any point without feeling obligated to continue. Personally identifiable data was not collected.

### 2.5. Ethics

The Bioethics Committee at the Medical University of Bialystok, Poland, approved the study (APK.002.587.2021).

### 2.6. Statistical Analyses

The statistical analysis was performed based on the Statistica 13.0 PL software. The correlations between variables were calculated using Spearman’s rank correlation analysis. The chi-square test was used for sample sizes five and larger. Fisher’s test was used for a sample size below 5. These tests were used to compare the percentages between countries. The Kruskal–Wallis test was used to compare the median values of scales. Statistically, significant differences and correlations were defined as *p* < 0.05.

## 3. Results

Table 1 illustrates the demographics of the respondents by status: not pregnant, pregnant, or postpartum. Among the surveyed women, the group of pregnant (8.1%) and postpartum women from Russia was very low (9.1%) and the highest from Poland (29.6% and 30.9%, respectively). Large differences were found in the residence structure of surveyed women from different countries, which probably depends on the geographical region where the surveys were conducted.

In Poland, respondents with a master’s degree dominated (201 people), and in Turkey with a bachelor’s degree (155 people). Most respondents from Belarus (114 people), Greece (116 people), and Russia (262 people) were students. Details are shown in Table 2.

Pregnant respondents had an average of 1.4 ± 0.7 children in Belarus, 1.4 ± 1.1 in Poland, 2.0 ± 0.9 in Greece, 3.1 ± 1.4 in Turkey, and 1.1 ± 1.2 in Russia. Postpartum respondents had an average of 1.7 ± 0.9 children in Belarus, 1.6 ± 0.9 in Poland, 2.1 ± 0.9 in Greece, 1.8 ± 1.5 in Turkey, and 1.8 ± 1.5 in Russia (Table 3).

Table 4 summarises information on the distribution of BDI measure values and risk of depression scores among pregnant women for each country. The differences between countries were very pronounced and statistically significant (*p* < 0.001). The highest risk of depression was shown in pregnant women from Turkey and Greece, and the lowest in Poland, although the difference for Belarusian or Russian women was small. A comparison of the severity of the risk of depression in postpartum women from the countries studied was made in a similar way. The pattern of results turned out to be similar to that for pregnant women—the highest risk was for Greek and Turkish women. The results were then contrasted, comparing BDI values within each country according to group association: not pregnant, pregnant, or postpartum. It was found that being pregnant or postpartum did not negatively affect the women’s mental health, which was evident in all countries. There were statistically significant differences in BDI levels (in Poland); the results suggest that in Poland, a significantly lower level of risk of depression affected ‘postpartum’ women (Table 4).

Among postpartum women, the level of depression was additionally examined using the EPDS questionnaire (Table 5). Significant differences were found in the level of depression among postpartum women from the studied countries. Women in Russia (5.9 points) scored most favourably, while Greece (9.3 points) scored worst.

The correlation between the two measures of the Beck scale (BDI) and the specific one targeting postpartum women’s experiences (EPDS) was tested. It was found that in all countries there was a statistically significant correlation between the specific EPDS measure and Beck’s overall depression measure: Belarus—correlation *r* = 0.50 (*p* < 0.001); Poland—correlation *r* = 0.60 (*p* < 0.001); Greece—correlation *r* = 0.44 (*p* < 0.001); Turkey—correlation *r* = 0.72 (*p* < 0.001), and Russia—correlation *r* = 0.653 (*p* < 0.001).

Significant differences in the distribution of the SWLS measure were found between pregnant women from the countries studied (Table 6). The highest life satisfaction was presented by pregnant women from Turkey and the lowest by pregnant women from Poland and Greece. In all countries, pregnant and postpartum women had higher life satisfaction, on average, by about 2–3 points (Table 6).

The GHQ-28 Questionnaire used to measure symptoms of mental breakdown in adults was used in further analysis. No significant differences in the severity of symptoms among the women from the studied countries were found (Table 7).

A measure of self-efficacy was also determined using the GSES questionnaire. No significant differences in the GSES among women from the assessed countries were noted.

In some countries, the sense of power necessary to initiate action in the KompOs scale was statistically different due to the woman’s family status. Women who had never been pregnant had a lower sense of power. Only Greek women who had never been pregnant had a higher sense of perseverance. The summary measure of personal competence showed more favourable, higher values for pregnant or postpartum women, apart from the Greek women (Table 7).

The way in which the mental state of pregnant women is affected by their age was studied. Two psychometric measures, BDI and SWLS, were included in this analysis. No significant correlations were found between age and risk of depression or satisfaction with life in the group of pregnant women, indicating that age is not a factor that would negatively affect the psychological well-being of pregnant women (Table S1).

An important issue that may affect the mental state of pregnant women seems to be a previous miscarriage (spontaneous and induced abortions). The women surveyed were, therefore, asked about their experience of spontaneous and induced abortions. No significant differences between countries in this matter were found. The results are shown in Table S2.

Table 8 assesses the correlation between BDI and SWLS and the experience of past miscarriage by women from each country using a chi-square test of independence. The differences between countries were statistically significant for the frequency of previous spontaneous abortions (highest in Turkey—32%, lowest in Belarus—13.9%). In none of the countries was there a statistically significant variation in the level of risk of depression among pregnant women in relation to the history of spontaneous abortion. Details are shown in Table 8.

Regarding both depression and satisfaction with life, having children was not shown to have an effect on the mental health of pregnant women. Details are shown in Table S3.

Analogously, a correlation was made between the duration of the last delivery and a measure of depression risk and satisfaction with life for pregnant women.

In Belarus, delivery lasted between ½ h and 17 h (5.8 ± 3.8 h); in Poland, between ½ h and 17 h (5.6 ± 3.6); in Greece, between 1 h and 42 h (10.8 ± 8.9 h); in Turkey, between 2 h and 18 h (4.6 ± 4.3 h), and in Russia between ½ h and 16 h (5.3 ± 4.2 h). A significant correlation between the number of children and BDI was found for women in Turkey (*r* = 0.21, *p* = 0.036). Details are shown in Table 9.

The influence of the type of recent delivery on the health of women who were pregnant at the time of the survey was also interesting. Respondents who had given birth previously or were in the postpartum period at the time of the survey were asked to specify the type of delivery. In the case of pregnant women in Belarus and Russia, natural childbirth predominated in Poland, Greece, and Russia. In contrast, in the postpartum group in Belarus, Poland, Greece, and Turkey, cesarean sections predominated. No significant differences were found in any of the countries analysed in the level of risk of depression or the level of life satisfaction among pregnant women in relation to the type of last delivery (Table 10).

The summary tables show Spearman’s rank correlation coefficients between the measures of mental health and quality of life and the GSES measure, as well as correlations with measures of personal competence. Due to the very large number of correlations analysed (the analysis was done by country and study group), exact *p*-test probability values were omitted, only signifying the level of statistical significance of a given correlation with a symbol. The tables use a colour scheme to facilitate interpretation of the results.

In most countries, the occurrence of negative mental states was correlated with poorer self-efficacy ratings, as evidenced by statistically significant negative correlations. Usually, these were weak correlations (absolute values of *r* are most often in the range of 0.30–0.50), but, for example, in Belarus and Russia, the mental health of pregnant women did not affect their self-efficacy ratings (the exception is the BDI measure, where correlations do occur). In Poland, the correlation of the measures considered with GSES was similar, regardless of the woman’s mental health in relation to being pregnant or having given birth. In Greece, women who were not pregnant showed no correlation between mental health and GSES (except for dysfunction and SWLS). The results are shown in Table 11.

The level of personal competence in terms of a sense of power was negatively correlated with greater severity of BDI and the presence of GHQ. The correlations are similar between countries; their strength is usually quite weak (coefficients in the range 0.30–0.50). Depressive states or mental disorders had the strongest effect on the sense of power in the Turkish community. However, some correlations were incidentally stronger (e.g., BDI and sense of power in Belarus or Russia). SWLS is positively correlated with a sense of power, and the strength of this correlation is similar to that of the BDI and GHQ measures.

The results are shown in Table 12.

The measures of depression, satisfaction with life, and especially the GHQ-28 were less strongly correlated with the sense of perseverance than was the case for the GSES measure or the sense of power (KompOs)—especially in the group of women from Poland and in Greece among non-pregnant women. Invariably, statistically significant correlations across all groups for all combinations of measures were found among Turkish women surveyed. Postpartum depression, according to the EPDS, was also the most severe, in terms of a sense of perseverance, among women from Turkey. The results are shown in Table 13.

The correlations between measures of depression, mental distress, and quality of life with the overall assessment of personal competence were quite pronounced, for some subgroups even more potent than any previous relationship (e.g., the correlation of SWLS and personal competence among postpartum Turkish women is *r* = 0.62, which is already quite high)—Table 14.

## 4. Discussion

It is worth mentioning that differences between the studied women from countries may exist according to cultural and educational factors. It is known that Eastern populations have been considered more family-centred and usually show more collective-oriented values, which tend to be more conservative and religious than others [18,19]. These factors might influence the social and familial support of pregnant women and how women experience maternity. In Turkey, 99% of the population is Islamic, 99% of the Polish population is Catholic, 95% of the Greek population is Orthodox, 72% of Russia’s population is Orthodox, and 48.3% of Belarus’s population is Orthodox [20], which makes these countries show some specific cultural characteristics. Culture has a significant influence on how depression manifests, is perceived, and is treated [21]. For example, some cultures might interpret symptoms of depression in a more somatic or physical way, focusing on complaints like fatigue or pain. In contrast, others may focus more on the emotional or cognitive symptoms. Furthermore, cultural factors can influence forms of therapy. Eastern cultures rely more on traditional medicines and practices than Western biological psychiatry. Understanding these cultural nuances in treating depression can help healthcare providers tailor treatment to the individual’s cultural background, potentially improving outcomes.

In the current study, the highest risk of depression was found in the comparative group and pregnant and postpartum women from Turkey and Greece and the lowest in Poland. Pregnant and postpartum women had by far the highest satisfaction with life in Turkey and the lowest in Greece. Mental health in the studied women was similar. The pregnant and postpartum women, except Greek women, had higher values of generalised self-efficacy. The risk of depression, the level of satisfaction with life, and the mental health of pregnant women were not influenced by the type of last delivery but by the duration of the last delivery.

Our findings are consistent with previous studies on the high incidence of depression among pregnant and non-pregnant women [8,9,10,18]. In the studied women from five countries, moderate symptoms of depression ranged from 10.7% to 25%. The highest risk of depression, according to the BDI, was found among pregnant women in Turkey and Greece and the lowest among pregnant women in Poland. Regarding the risk of depression and life satisfaction, having children was not found to affect the mental health of pregnant women. Only in Turkey did pregnant women without children have a slightly higher quality of life, and the number of children that a pregnant woman had negatively affected her mental health.

The prevalence of mood disorders during pregnancy and the postpartum period is thought to have increased significantly in the last decade. The estimated incidence of depression across different stages of life fluctuates between 14.4% and 18.0% [18]. When Khamees et al. [22] assessed anxiety and depression among 120 pregnant women using the EPDS, 44.2% of women reported experiencing depression. In 2017, 5.3% of women in the European Union were also found to suffer from depression, with the highest rates in Portugal and Finland [6.5%] and a rate of 3.2% in Poland [23]. In Durankuş and Aksu’s [24] study, 35.4% of pregnant women (*n* = 92) scored higher than 13 on the EPDS and indicated the statistically significant effect of COVID-19 on their mean BDI scores. Meanwhile, among postpartum women, the greatest risk of depression was for women in Greece and Turkey. Being pregnant or in the postpartum period did not negatively affect women’s mental health, as was evident in all countries.

Although PPD affects approximately 7–20% of women [25,26,27], up to an estimated 50% of women who suffer from PPD do not see a doctor or are not asked about whether they are experiencing symptoms of mood disorders. Left untreated, PPD can induce adverse changes in a woman’s relationship with her child, partner, and family and can have a negative, distancing effect on the child’s emotional development. In our study, PDD affected 7.6–37.4% of women; the greatest risk of postpartum depression was for women in Turkey.

In a Polish study [28], mild depressive states occurred in 11.96% of patients in the first week after childbirth and, by 30 days after childbirth, were observed in 19.51% of patients. The most common predisposing factors for mood disorders related to childbirth were unplanned pregnancy, health complications during pregnancy, and hospitalisation due to a high-risk pregnancy.

Satisfaction with particular elements of life is characterised by intense feelings that emerge after a prolonged period of contentment, defined as a temporary feeling of satisfaction.

In Kazmierczak et al.’s [18] study, which included 100 women in the third trimester of pregnancy, the mean SWLS score was 23.21, indicating a high life satisfaction level in the sample overall. However, the degree of life satisfaction among the women was not differentiated by age, level of education, occupational activity, place of residence, economic status, parity, duration of previous pregnancy, type of pregnancy ending, course of current pregnancy, illness in pregnancy, or hospitalisation during the current pregnancy. Meanwhile, in Kanadys et al.’s [29] study, 70% of pregnant women had moderate life satisfaction, 14.28% had a high level, and 14.78% had a low level. Women’s level of life satisfaction did not change significantly in relation to age in the studies by Kanadys et al. [29], Majda et al. [30], or Jachimowicz et al. [31].

In our study, mean SWLS scores were generally higher than in Kazmierczak et al.’s [18] and Kandys et al.’s [29] studies, which showed by far the highest scores for satisfaction obtained by pregnant women in Turkey and the lowest obtained by pregnant women in Poland and Greece.

In Kazmierczak et al.’s [18] study, childbearing differentiated life satisfaction among pregnant women, such that primiparous women scored higher on the SWLS than multiparous ones. At the same time, Kalecińska-Adamczyk and Serafińska [32] reported that having one child was conducive to a more positive assessment of quality of life than having multiple or none. Women without children rated their quality of life as being significantly lower in the psychosocial domain than in other domains, while having multiple children or none at all had similar effects on the sense of the quality of life in the psychophysical and subjective domains. Those trends were confirmed by our results, pregnant and postpartum women in all countries had higher life satisfaction than non-pregnant and childless women.

In the present study, the GSES was used to measure the strength of the women’s self-efficacy in coping with difficult situations and obstacles. In our study, neither being pregnant nor being in the postpartum period had a depressive effect on women’s self-efficacy in any of the culturally distinct communities studied. In most countries, the presence of poor mental health was correlated with poorer self-efficacy.

In Rogala and Ossowski’s [33] study, women with normal pregnancies reported a high level of general self-efficacy, at 30.19, compared with the Polish average of 27.32. Moreover, the level of self-efficacy did not seem to correlate with the duration of delivery, the activity of the woman giving birth, the number of analgesics used, or the method of delivery.

In Kamasz et al.’s [34] study, conducted in young women aged 19–24 years old who had never been pregnant, most of the women were moderately or severely afraid of childbirth, especially complications and pain associated with childbirth. More than 75% of the women surveyed had heard of traumatic birth experiences from their relatives. Even so, nearly 92% of the women surveyed indicated desiring to become pregnant and give birth, and more than 90% perceived childbirth as a natural phenomenon. One factor, namely traumatic narratives about childbirth in the environment or family, was found to influence GSES scores.

Using the KompOs scale, we also examined women’s sense of competence, with a special focus on subjective confidence in overcoming difficulties and situational adversities and in achieving a set goal. Our study found that, in most communities, but not Russia, the sense of the power needed to initiate action was differentiated by the woman’s status in the family, such that women who had never been pregnant had a lower sense of power. A similar pattern surfaced among women in Turkey and Russia in their sense of perseverance; only women in Greece who had never been pregnant were distinguished by a higher sense of perseverance.

Across all countries examined, the level of personal competence within the sense of power was negatively correlated with higher severity of depression and the presence of psychological distress. Depressive states or psychological disorders had the strongest effect on the sense of power among women in Turkey.

When we used the GHQ-28 to assess participating women’s mental health, no significant differences arose in the severity of symptoms between the three groups compared with any of the study’s various populations. The measures of depression, life satisfaction, and especially the GHQ-28 were less strongly correlated with a sense of perseverance than GSES score or sense of power on KompOs, especially among women in Poland and among non-pregnant women in Greece.

Among other results, in no country analysed did differences emerge in the level of the risk of depression or life satisfaction among pregnant women relative to the type of previous delivery. Thus, that factor did not seem to affect the psychological well-being of the pregnant women.

An unpredictable event that cannot be adequately prepared for regardless of the circumstances is a high-risk pregnancy or miscarriage [35].

In our study, we found fairly large differences between countries in the correlation between BDI and SWLS scores and experiences with spontaneous abortion—differences were largest in Turkey and smallest in Belarus. However, none of the countries showed significant variation in the level of the risk of depression for pregnant women in relation to their history of spontaneous abortion. Nevertheless, in Poland and Belarus, the risk of depression was higher for women who had undergone an induced abortion.

An analysis of epidemiological data purporting the frequency of miscarriages revealed that between 10% and 24% of all pregnancies end in miscarriage [36]. Psychological consequences of experiencing miscarriage include increased risk of anxiety, depression, post-traumatic stress disorder, and suicide [37], and the most common mental states are shock, denial, anger, rage, guilt, and helplessness, the strength of each of which is stimulated by the meaning given to the lost pregnancy [38].

Based on their research among women and parents experiencing miscarriage, Neugebauer [39] and Brier [40] have emphasised the extraordinary intensity of the psychological reactions at the time of the event and noted the long-term impact of the experience’s negative effects on mental health.

Depression may be associated with a lower likelihood of having children. A nationwide register study in Finland [41] examined associations between depression and the likelihood of having children, the number of children, and the parental age at first birth. Women with secondary care-treated depression have a lower likelihood of having children and have fewer children. They also found that depression was associated with a slightly lower parental age at first birth.

### Limitations of the Study

The group of pregnant and postpartum women in Russia was relatively small, which may have limited the possibility of comparisons between them and women from other countries. Due to differences in the residence structure of women respondents from different countries depending on the region where the surveys were conducted, we assumed that, in the 21st century, place of residence does not significantly determine the quality of life, and its influence should not be considered further. In Russia, Belarus, and, to a slightly lesser extent, Greece, the surveys of non-pregnant women were conducted primarily among young people, which may have significantly influenced the results. However, the literature contains no studies of a similar scope that would allow us to compare our results with other research findings.

## 5. Implications

Women are twice as likely to develop depression, and the time of first onset is often the perinatal period. We suggest that pregnant women should be screened for depression (Polish guidelines) at least three times during the first trimester, mid-pregnancy, and postpartum. Early detection of depressive symptoms and implementation of treatment reduces the risk of developing a major depressive episode and has a preventive effect on suicidal behavior. Educational activities should be carried out on depression in pregnant and postpartum women among partners, families, midwives, gynaecologists, and family doctors.

## 6. Conclusions

The highest risk of depression was found in the control group and pregnant and postpartum women from Turkey and Greece and the lowest in Poland. Pregnant and postpartum women had by far the highest satisfaction with life in Turkey and the lowest in Greece. The efficacy in dealing with difficult situations was similar in all studied countries. Also, mental health according to the GHQ-28 in studied women was similar. The pregnant and postpartum women, except Greek women, had higher values of generalised self-efficacy on the KompOs scale. The risk of depression, the level of satisfaction with life, and the mental health of pregnant women were not influenced by the type of last delivery but by the duration of the last delivery.

## Figures and Tables

**Table 1:** Demographics of the respondents by status: not pregnant, pregnant, postpartum.

**Group**		**Country—Group Size**						
		**Belarus**		**Poland**	**Greece**		**Turkey**	**Russia**
Control *N* = 906		136 (15.0%)		131 (14.5%)	144 (15.9%)		150 (16.6%)	345 (38.0%)
Pregnant *N* = 584		147 (25.2%)		173 (29.6%)	114 (19.5%)		103 (17.6%)	47 (8.1%)
Postpartum *N* = 527		103 (19.5%)		163 (30.9%)	106 (20.1%)		107 (20.3%)	48 (9.1%)
Total *N* = 2017		386 (19.1%)		467 (23.2%)	364 (18.0%)		360 (17.8%)	440 (21.9%)
**Group**	**Country**	**Age [Years]**						
		$\bar{x}$	**Me**	***s***	***c*_25_**	***c*_75_**	**Min.**	**Max.**
Control	Belarus	19.2	19	2.0	18	20	17	30
	Poland	33.5	33	9.0	26	39	18	50
	Greece	21.0	20	4.0	19	21	18	50
	Turkey	28.3	26	8.1	22	33	17	49
	Russia	18.5	18	1.1	18	19	17	24
Pregnant	Belarus	29.2	29	5.5	25	33	18	40
	Poland	30.4	30	5.0	27	34	20	43
	Greece	34.7	33	8.9	27	43	19	50
	Turkey	27.7	27	5.3	24	31	17	41
	Russia	27.7	29	4.6	24	30	17	37
Postpartum	Belarus	28.7	29	5.3	25	32	18	42
	Poland	31.0	30	5.4	27	35	20	45
	Greece	37.6	36	8.8	30	45	19	50
	Turkey	28.1	28	4.7	25	31	19	44
	Russia	28.1	28	4.3	24.5	31.5	18	37
	**Place of Residence**							**Total** ***N* = 2017**
	**Belarus** ***N* = 386**	**Poland** ***N* = 467**		**Greece** ***N* = 364**	**Turkey** ***N* = 360**		**Russia** ***N* = 440**	
rural	5.8%	33.5%		28.1%	16.7%		7.6%	18.0%
City with a population <5000	2.9%	6.1%		12.8%	0.0%		5.3%	5.3%
City with a population of 5000–25,000	10.6%	12.9%		11.9%	0.3%		10.1%	9.2%
City with a population of 25,000–40,000	4.0%	10.7%		8.0%	19.8%		2.7%	8.7%
City with a population of 40,000–100,000	4.5%	13.6%		12.5%	26.2%		6.9%	12.2%
City with a population of 100,000–200,000	4.2%	3.5%		4.8%	8.9%		5.9%	5.3%
City with a population >200,000	68.0%	19.7%		21.9%	28.1%		61.6%	39.4%
No answer	2.0%	2.1%		3.3%	0.3%		0.7%	1.7%

Notes $\bar{x}$—mean, Me—Median.

**Table 2:** Educational background of respondents.

Education	Belarus *N* = 386			Poland *N* = 467			Greece *N* = 364			Turkey *N* = 360			Russia *N* = 440			Total
	1	2	3	1	2	3	1	2	3	1	2	3	1	2	3	
	*N* = 136	*N* = 147	*N* = 103	*N* = 131	*N* = 173	*N* = 163	*N* = 144	*N* = 114	*N* = 106	*N* = 150	*N* = 103	*N* = 107	*N* = 345	*N* = 47	*N* = 48	
vocational	4	41	37	7	11	11	1	18	15	51	36	42	5	16	22	317
Total	82			29			34			129			43			
bachelor’s degree	2	35	16	33	28	23	27	33	32	74	40	41	4	12	8	408
Total	53			84			92			155			24			
master’s degree	9	40	23	42	75	84	3	5	8	18	8	11	9	6	13	354
Total	72			201			16			37			28			
student	92	12	10	14	10	13	109	3	4	1	1	13	250	10	2	544
Total	114			37			116			15			262			
secondary education	29	19	17	35	49	32	4	55	47	6	18	0	77	3	3	394
Total	65			116			106			24			83			

1—Control group; 2—pregnant women; 3—postpartum women.

**Table 3:** Number of children.

Number of Children	Belarus *N* = 386			Poland *N* = 467			Greece *N* = 364			Turkey *N* = 360			Russia *N* = 440		
	1	2	3	1	2	3	1	2	3	1	2	3	1	2	3
	*N* = 136	*N* = 147	*N* = 103	*N* = 131	*N* = 173	*N* = 163	*N* = 144	*N* = 114	*N* = 106	*N* = 150	*N* = 103	*N* = 107	*N* = 345	*N* = 47	*N* = 48
Min.	0	0	0	0	0	0	0	0	0	0	0	0	0	0	0
Max.	0	3	5	0	5	5	0	5	5	0	5	5	0	5	5
Average number of children	0	1.4 ± 0.7	1.7 ± 0.9	0	1.4 ± 1.1	1.6 ± 0.9	0	2.0 ± 0.9	2.1 ± 0.9	0	3.1 ± 1.4	1.8 ± 1.5	0	1.1 ± 1.2	1.8 ± 1.0

**Table 4:** Risk of depression scores among pregnant and postpartum women.

**Pregnant Women**															
**Country**	**Beck Depression Inventory (*p* < 0.001)**														
	**Mean (95% c.i.)**					**Me**	***s***			***c*** **_25_**			***c*** **_75_**		
Belarus (*N* = 147)	6.2 (5.4–7.0)					5	4.8			2.5			9		
Poland (*N* = 173)	5.9 (5.0–6.9)					4	6.4			1			8.5		
Greece (*N* = 114)	8.3 (7.1–9.6)					8	6.6			4			11		
Turkey (*N* = 103)	8.4 (7.5–9.4)					8	5.0			5			12		
Russia (*N* = 48)	7.6 (5.9–9.3)					6	5.5			4			10		
*p*—test probability values calculated using the Kruskal–Wallis test															
**Country**	**Risk of Depression (*p* = 0.020)**														
	**no**						**moderate**						**severe**		
Belarus (*N* = 147)	84.3%						15.7%						0.0%		
Poland (*N* = 173)	86.9%						10.7%						2.4%		
Greece (*N* = 114)	76.1%						22.0%						1.8%		
Turkey (*N* = 103)	74.8%						25.2%						0.0%		
Russia (*N* = 47)	77.8%						22.2%						0.0%		
*p*—test probability values calculated using the chi-square test of independence															
**Postpartum Women**															
**Country**	**Beck Depression Inventory (*p* < 0.001)**														
	$\bar{x}$						**Me**		***s***		***c*** **_25_**		***c*** **_75_**		
Belarus (*N* = 103)	5.3						5		4.4		2		7		
Poland (*N* = 163)	4.9						2		6.7		0		6		
Greece (*N* = 106)	8.4						8		7.9		3		11		
Turkey (*N* = 107)	10.8						10		7.2		5		15		
Russia (*N* = 48)	5.3						5		4.9		2		8		
***p*** **—test probability values calculated using the Kruskal–Wallis test**															
**Country**	**Risk of Depression (*p* ≤ 0.001)**														
	**No**						**Moderate**						**Severe**		
Belarus (*N* = 103)	92.4%						7.6%						0.0%		
Poland (*N* = 163)	87.4%						10.7%						1.9%		
Greece (*N* = 106)	75.7%						22.3%						1.9%		
Turkey (*N* = 107)	59.8%						37.4%						2.8%		
Russia (*N* = 48)	87.2%						12.8%						0.0%		
*p*—test probability values calculated using the chi-square test of independence and Fisher’s test															
**Comparison of All Study Groups**															
**Group**	**Beck Depression Inventory**														
	**Belarus**			**Poland**			**Greece**			**Turkey**			**Russia**		
	$\bar{x}$	**Me**	**IQR**	$\bar{x}$	**Me**	**IQR**	$\bar{x}$	**Me**	**IQR**	$\bar{x}$	**Me**	**IQR**	$\bar{x}$	**Me**	**IQR**
Control	6.5	4	9	5.8	3	8	9.8	8	10	11.1	10	12	7.5	5	9
pregnant	6.2	5	6.5	5.9	4	7.5	8.3	8	7	8.4	8	7	7.6	6	6
postpartum	5.3	5	5	4.9	2	6	8.4	8	8	10.8	10	10	5.3	5	6
*p*	0.2500			0.0158 *			0.4165			0.0812			0.1249		
*p*—test probability values calculated using the Kruskal–Wallis test															

Notes $\bar{x}$—Mean, Me—Median, IQR—interquartile range, * *p* < 0.05.

**Table 5:** Distribution of EPDS measure values among women from each country.

Country	(*p* < 0.001)				
	Mean (95% c.i.)	Me	*s*	*c*_25_	*c*_75_
Belarus	6.2 (5.5–6.9)	6	3.7	4	9
Poland	7.6 (6.7–8.4)	6	5.8	3	10
Greece	9.3 (8.2–10.4)	9	5.6	6	12
Turkey	9.2 (8.2–10.2)	8	5.2	5	13
Russia	5.9 (4.8–7.0)	5	3.7	3	8

*p*—test probability values calculated using the Kruskal–Wallis test. Me—Median.

**Table 6:** Assessment of women’s satisfaction with life.

**Pregnant Women**															
**Country**				**SWLS Scale (*p* < 0.001)**											
				**Mean (95% c.i.)**				**Me**		***s***		***c*** **_25_**		***c*** **_75_**	
Belarus				26.4 (25.6–27.2)				27		4.8		23		30	
Poland				25.2 (24.4–26.0)				25		5.2		22		29	
Greece				25.1 (23.9–26.2)				26		6.2		20.5		30	
Turkey				28.6 (27.8–29.4)				29		4.0		26		30	
Russia				26.3 (24.7–27.8)				27		5.2		25		30	
*p*—test probability values calculated using the Kruskal–Wallis test															
				**SWLS Adjective Scale (*p* < 0.001)**											
				**Belarus**			**Poland**			**Greece**		**Turkey**		**Russia**	
extremely dissatisfied				0.0%			0.6%			1.8%		0.0%		0.0%	
very dissatisfied				0.0%			2.4%			2.7%		1.0%		0.0%	
slightly dissatisfied				7.2%			9.5%			14.3%		0.0%		17.8%	
neutral				8 5.8%			4.8%			6.3%		2.9%		0.0%	
slightly satisfied				28.8%			34.5%			20.5%		18.4%		13.3%	
very satisfied				39.6%			33.9%			34.8%		53.4%		53.3%	
extremely satisfied				18.7%			14.3%			19.6%		24.3%		15.6%	
*p*—test probability values calculated using the chi-square test of independence and Fisher’s test															
POSTPARTUM WOMEN															
**Country**				**SWLS Scale (*p* = 0.056)**											
				**Mean (95% c.i.)**				**Me**		***s***		***c*** **_25_**		***c*** **_75_**	
Belarus				26.2 (25.2–27.2)				26		4.9		23.5		29.5	
Poland				26.7 (25.9–27.4)				27		4.7		24		30	
Greece				24.6 (23.4–25.9)				25		6.5		21		29	
Turkey				26.8 (25.9–27.7)				27		4.6		25		30	
Russia				26.3 (25.0–27.7)				27		4.3		23		29	
*p*—test probability values calculated using the Kruskal–Wallis test															
				**SWLS Adjective Scale (*p* = 0.136)**											
				**Belarus** ***N* = 92**			**Poland** ***N* = 156**			**Greece** ***N* = 104**		**Turkey** ***N* = 107**		**Russia** ***N* = 43**	
extremely dissatisfied				0 (0.0%)			0 (0.0%)			2 (1.9%)		0 (0.0%)		0 (0.0%)	
very dissatisfied				3 (3.3%)			2 (1.3%)			7 (6.7%)		1 (0.9%)		0 (0.0%)	
slightly dissatisfied				3 (3.3%)			9 (5.8%)			8 (7.7%)		9 (8.4%)		2 (4.7%)	
neutral				2 (2.2%)			5 (3.2%)			5 (4.8%)		2 (1.9%)		1 (2.3%)	
slightly satisfied				35 (38.0%)			42 (26.9%)			31 (29.8%)		27 (25.2%)		18 (41.9%)	
very satisfied				33 (35.9%)			68 (43.6%)			34 (32.7%)		49 (45.8%)		15 (34.9%)	
extremely satisfied				16 (17.4%)			30 (19.2%)			17 (16.3%)		19 (17.8%)		7 (16.3%)	
*p*—test probability values calculated using the chi-square test of independence, and and Fisher’s test															
**Comparison of All Study Groups**															
**Group**	**SWLS Scale**														
	**Belarus**			**Poland**			**Greece**			**Turkey**			**Russia**		
	$\bar{x}$	**Me**	**IQR**	$\bar{x}$	**Me**	**IQR**	$\bar{x}$	**Me**	**IQR**	$\bar{x}$	**Me**	**IQR**	$\bar{x}$	**Me**	**IQR**
Control	23.5	24	8.0	24.0	24	6.0	23.9	24	6.0	23.3	25	10.0	23.9	24	10.0
pregnant	26.4	27	7.0	25.2	25	7.0	25.1	26	9.5	28.6	29	4.0	26.3	27	5.0
postpartum	26.2	26	6.0	26.7	27	6.0	24.6	25	8.0	26.8	27	5.0	26.3	27	6.0
*p*	<0.001			<0.001			0.1725			<0.001			0.0048		
*p*—test probability values calculated using the Kruskal–Wallis test															

Notes $\bar{x}$—Mean, Me—Median, IQR—interquartile range.

**Table 7:** Distribution of GHQ-28, GSES, and KompOs measure values among women from each country.

**Group**	**GHQ-28 (Summary Measure)**														
	**Belarus**			**Poland**			**Greece**			**Turkey**			**Russia**		
	$\bar{x}$	**Me**	**IQR**	$\bar{x}$	**Me**	**IQR**	$\bar{x}$	**Me**	**IQR**	$\bar{x}$	**Me**	**IQR**	$\bar{x}$	**Me**	**IQR**
Control	5.4	4	8.0	5.3	3	9.0	5.8	5	7.5	4.9	2	9.0	5.4	3	9.0
pregnant	5.3	5	7.0	4.4	2	8.0	5.4	4	7.0	3.9	2	6.0	6.0	5.5	7.0
postpartum	4.8	4	7.0	4.7	3	8.0	5.2	4	7.0	5.0	2	6.0	5.6	5.5	6.0
*p*	0.8209			0.9540			0.4731			0.1882			0.1378		
**Group**	**GSES**														
	**Belarus**			**Poland**			**Greece**			**Turkey**			**Russia**		
	$\bar{x}$	**Me**	**IQR**	$\bar{x}$	**Me**	**IQR**	$\bar{x}$	**Me**	**IQR**	$\bar{x}$	**Me**	**IQR**	$\bar{x}$	**Me**	**IQR**
Control	30.4	32	10.0	31.4	31	4.0	31.8	32	5.0	27.2	27	11.0	30.2	31	9.0
pregnant	30.7	31	8.0	31.6	31	4.0	32.3	33	6.0	27.0	27	8.0	31.2	32	7.0
postpartum	30.9	33	8.0	31.7	31	5.0	32.7	33	6.0	27.2	27	9.0	32.2	32	6.0
*p*	0.9364			0.6612			0.3941			0.9584			0.4451		
**Group**	**KompOs—Sense of Power**														
	**Belarus**			**Poland**			**Greece**			**Turkey**			**Russia**		
	$\bar{x}$	**Me**	**IQR**	$\bar{x}$	**Me**	**IQR**	$\bar{x}$	**Me**	**IQR**	$\bar{x}$	**Me**	**IQR**	$\bar{x}$	**Me**	**IQR**
Control	18.7	19	5.0	17.5	18	4.0	17.8	18	4.0	16.9	17	6.0	19.2	20	3.0
pregnant	19.8	20	3.5	18.3	18	3.0	18.6	19	5.0	18.4	19	5.0	19.8	20	3.0
postpartum	19.3	20	3.0	18.2	18	3.0	18.5	18	5.0	17.8	19	7.0	19.8	19	4.0
*p*	0.0574			0.0210 *			0.0979			0.0124 *			0.5798		
**Group**	**KompOs—Sense of Perseverance**														
	**Belarus**			**Poland**			**Greece**			**Turkey**			**Russia**		
	$\bar{x}$	**Me**	**IQR**	$\bar{x}$	**Me**	**IQR**	$\bar{x}$	**Me**	**IQR**	$\bar{x}$	**Me**	**IQR**	$\bar{x}$	**Me**	**IQR**
Control	18.0	19	5.0	17.3	16.5	5.0	19.1	19	4.0	17.2	17	5.0	17.6	17	5.0
pregnant	18.3	18	5.0	17.9	18	5.0	17.8	18	6.0	18.3	19	6.0	18.5	18	5.0
postpartum	18.2	18	5.0	17.7	17	5.0	18.2	18	5.0	18.4	18	5.0	18.4	18	4.0
*p*	0.8479			0.1459			0.0234 *			0.0089 **			0.0970		
**Group**	**KompOs—Summary measure of personal competence**														
	**Belarus**			**Poland**			**Greece**			**Turkey**			**Russia**		
	$\bar{x}$	**Me**	**IQR**	$\bar{x}$	**Me**	**IQR**	$\bar{x}$	**Me**	**IQR**	$\bar{x}$	**Me**	**IQR**	$\bar{x}$	**Me**	**IQR**
Control	36.7	37	8.0	34.9	34	7.0	36.9	37	8.0	34.1	34	9.0	36.8	37	6.0
pregnant	38.1	38	7.5	36.2	36	5.0	36.4	36	9.0	36.7	37	9.0	38.3	37	6.0
postpartum	37.5	38	6.0	35.9	35	6.0	36.7	37	9.0	36.2	38	11.0	38.3	38	7.0
*p*	0.1721			0.0244 *			0.9106			0.0019 **			0.1002		

Notes $\bar{x}$—Mean, Me—Median, IQR- interquartile range, * *p* < 0.05, ** *p* < 0.01.

**Table 8:** Correlations between BDI and SWLS and a history of miscarriage among women from each country.

**Country**				**History of Spontaneous Abortions**						**History of Induced Abortions**					
				**%**						**%**					
Belarus				13.9%						11.8%					
Poland				20.9%						3.7%					
Greece				22.5%						10.2%					
Turkey				32.0%						11.7%					
Russia				26.2%						9.8%					
				*p* = 0.0160 * Chi-square test of indepedence						0.0894					
**Beck Depression Inventory vs. History of Spontaneous Abortions**															
	**Belarus**			**Poland**			**Greece**			**Turkey**			**Russia**		
	$\bar{x}$	**Me**	**IQR**	$\bar{x}$	**Me**	**IQR**	$\bar{x}$	**Me**	**IQR**	$\bar{x}$	**Me**	**IQR**	$\bar{x}$	**Me**	**IQR**
yes	6.5	7	8.0	4.4	4	5.0	6.9	6	8.0	8.8	8	6.0	8.8	9	8.0
no	6.2	5	6.0	5.9	5	8.0	8.8	8	8.0	8.3	8.5	7.0	7.2	5	5.0
*p*	0.9462			0.3780			0.3242			0.8522			0.2294		
**SWLS vs. History of Spontaneous Abortions**															
	**Belarus**			**Poland**			**Greece**			**Turkey**			**Russia**		
	$\bar{x}$	**Me**	**IQR**	$\bar{x}$	**Me**	**IQR**	$\bar{x}$	**Me**	**IQR**	$\bar{x}$	**Me**	**IQR**	$\bar{x}$	**Me**	**IQR**
yes	25.3	24	8.0	25.8	26	7.0	24.3	25	10.0	29.0	29	5.0	24.7	26	9.0
no	26.7	27	7.0	25.2	25	7.0	25.3	26	9.0	28.4	29	4.0	26.8	27	5.0
*p*	0.2743			0.6548			0.3666			0.5197			0.3225		
**Beck Depression Inventory vs. History of Induced Abortions**															
	**Belarus**			**Poland**			**Greece**			**Turkey**			**Russia**		
	$\bar{x}$	**Me**	**IQR**	$\bar{x}$	**Me**	**IQR**	$\bar{x}$	**Me**	**IQR**	$\bar{x}$	**Me**	**IQR**	$\bar{x}$	**Me**	**IQR**
yes	8.7	9	11.0	10.0	7	8.0	8.8	7	12.0	8.6	9.5	10.0	7.0	4.5	6.0
no	5.9	5	5.0	5.6	4	7.0	8.2	7.5	7.0	8.4	8	6.0	7.7	7	7.0
*p*	0.0849			0.7108			0.8648			0.7723			0.9822		
**SWLS vs. History of Induced Abortions**															
	**Belarus**			**Poland**			**Greece**			**Turkey**			**Russia**		
	$\bar{x}$	**Me**	**IQR**	$\bar{x}$	**Me**	**IQR**	$\bar{x}$	**Me**	**IQR**	$\bar{x}$	**Me**	**IQR**	$\bar{x}$	**Me**	**IQR**
yes	24.6	23	7.0	26.0	27	8.0	24.9	25	11.0	27.9	26.5	6.0	25.3	25	10.5
no	26.7	27	7.0	25.2	25	7.0	25.2	26	9.0	28.7	29	4.0	26.6	27	5.0
*p*	0.1004			0.6008			0.7637			0.2384			0.4879		

Notes $\bar{x}$—Mean, Me—Median, IQR—interquartile range, * *p* < 0.05.

**Table 9:** Correlations between BDI and SWLS and delivery duration in women from each country.

Pregnant Women		
Country	Number of Children vs. BDI	Number of Children vs. SWLS
Belarus	*r* = 0.02 (*p* = 0.7804)	*r* = −0.14 (*p* = 0.0942)
Poland	*r* = −0.03 (*p* = 0.7212)	*r* = −0.02 (*p* = 0.7644)
Greece	*r* = −0.02 (*p* = 0.8172)	*r* = −0.10 (*p* = 0.2959)
Turkey	*r* = 0.21 (*p* = 0.0360 *)	*r* = −0.25 (*p* = 0.0121 *)
Russia	*r* = −0.09 (*p* = 0.5541)	*r* = −0.10 (*p* = 0.4952)
	**Delivery Duration vs. BDI**	**Delivery Duration vs. SWLS**
Belarus	*r* = 0.25 (*p* = 0.0709)	*r* = −0.10 (*p* = 0.4867)
Poland	*r* = −0.12 (*p* = 0.3584)	*r* = −0.10 (*p* = 0.4462)
Greece	*r* = 0.00 (*p* = 0.9923)	*r* = 0.09 (*p* = 0.4737)
Turkey	*r* = −0.20 (*p* = 0.2021)	*r* = −0.02 (*p* = 0.8774)
Russia	*r* = −0.14 (*p* = 0.6109)	*r* = −0.25 (*p* = 0.3418)

Notes *r* = Spearman correlation coefficient, * *p* < 0.05.

**Table 10:** Influence of type of last delivery on risk of depression and life satisfaction among women from the countries concerned.

**Type of Delivery (*p* = 0.0121)**															
	**Belarus**			**Poland**			**Greece**			**Turkey**			**Russia**		
Natural	66.7%			60.2%			67.7%			39.5%			76.5%		
Caesarean section	33.3%			39.8%			32.3%			60.5%			23.5%		
Total	75			88			93			43			17		
**Beck Depression Inventory vs. Type of Delivery**															
	**Belarus**			**Poland**			**Greece**			**Turkey**			**Russia**		
	$\bar{x}$	**Me**	**IQR**	$\bar{x}$	**Me**	**IQR**	$\bar{x}$	**Me**	**IQR**	$\bar{x}$	**Me**	**IQR**	$\bar{x}$	**Me**	**IQR**
Natural	6.5	6	7.0	6.0	4	8.0	9.0	8.5	9.0	8.5	9	7.0	6.1	4	5.0
c.s.	6.4	4	12.0	5.2	5	7.0	6.7	7	6.0	9.6	9	6.0	10.3	8.5	9.5
*p*	0.7303			0.9821			0.2207			0.5801			0.2958		
**SWLS vs. Type of Delivery**															
	**Belarus**			**Poland**			**Greece**			**Turkey**			**Russia**		
	$\bar{x}$	**Me**	**IQR**	$\bar{x}$	**Me**	**IQR**	$\bar{x}$	**Me**	**IQR**	$\bar{x}$	**Me**	**IQR**	$\bar{x}$	**Me**	**IQR**
Natural	25.8	26	7.0	25.2	26	8.0	25.2	26	9.0	27.6	29	5.0	25.6	26	9.0
c.s.	26.7	27.5	7.0	24.4	25	6.0	25.2	26.5	9.0	27.7	27	6.0	24.8	26	5.5
*p*	0.4155			0.5578			0.9967			0.9706			0.7034		

Notes $\bar{x}$—Mean, Me—Median, IQR—interquartile range.

**Table 11:** Correlations between GSES and other psychometric measures.

Measures of Depression, Mental Distress and Quality of Life	GSES and Other Psychometric Measures				
	Belarus	Poland	Greece	Turkey	Russia
Not pregnant women					
BDI	*r* = −0.37 ***	*r* = −0.42 ***	*r* = −0.20	*r* = −0.48 ***	*r* = −0.40 ***
SWLS	*r* = 0.41 ***	*r* = 0.44 ***	*r* = 0.22 *	*r* = 0.31 ***	*r* = 0.37 ***
GHQ (somatic symptoms)	*r* = −0.18 *	*r* = −0.20 *	*r* = 0.00	*r* = −0.28 ***	*r* = −0.26 ***
GHQ (anxiety, insomnia)	*r* = −0.18 *	*r* = −0.18 *	*r* = −0.11	*r* = −0.35 ***	*r* = −0.30 ***
GHQ (dysfunctions)	*r* = −0.30 ***	*r* = −0.19 *	*r* = −0.31 ***	*r* = −0.31 ***	*r* = −0.35 ***
GHQ (depressive symptoms)	*r* = −0.23 **	*r* = −0.20 *	*r* = −0.09	*r* = −0.25 **	*r* = −0.26 ***
GHQ (total score)	*r* = −0.23 **	*r* = −0.22 *	*r* = −0.13	*r* = −0.37 ***	*r* = −0.35 ***
Pregnant women					
BDI	*r* = −0.27 **	*r* = −0.42 ***	*r* = −0.39 ***	*r* = −0.51 ***	*r* = −0.57 ***
SWLSr	*r* = 0.11	*r* = 0.33 ***	*r* = 0.43 ***	*r* = 0.43 ***	*r* = 0.24
GHQ (somatic symptoms)	*r* = −0.07	*r* = −0.18 *	*r* = −0.27 **	*r* = −0.33 ***	*r* = 0.00
GHQ (anxiety, insomnia)	*r* = −0.10	*r* = −0.18 *	*r* = −0.34 ***	*r* = −0.31 **	*r* = −0.15
GHQ (dysfunctions)	*r* = −0.05	*r* = −0.24 **	*r* = −0.32 ***	*r* = −0.31 **	*r* = −0.03
GHQ (depressive symptoms)	*r* = −0.07	*r* = −0.27 ***	*r* = −0.26 **	*r* = −0.24 *	*r* = −0.13
GHQ (total score)	*r* = −0.10	*r* = −0.25 **	*r* = −0.37 ***	*r* = −0.42 ***	*r* = −0.08
Postpartum women					
BDI	*r* = −0.18	*r* = −0.49 ***	*r* = −0.42 ***	*r* = −0.45 ***	*r* = −0.57 ***
EPDS	*r* = −0.30 **	*r* = −0.42 ***	*r* = −0.41 ***	*r* = −0.39 ***	*r* = −0.39 **
SWLS	*r* = 0.38 ***	*r* = 0.37 ***	*r* = 0.40 ***	*r* = 0.33 ***	*r* = 0.30
GHQ (somatic symptoms)	*r* = −0.07	*r* = −0.18 *	*r* = −0.25 *	*r* = −0.38 ***	*r* = −0.10
GHQ (anxiety, insomnia)	*r* = −0.19	*r* = −0.05	*r* = −0.37 ***	*r* = −0.24 *	*r* = −0.20
GHQ (dysfunctions)	*r* = −0.18	*r* = −0.29 ***	*r* = −0.30 **	*r* = −0.42 ***	*r* = −0.38 *
GHQ (depressive symptoms)	*r* = −0.17	*r* = −0.18 *	*r* = −0.33 ***	*r* = −0.33 ***	*r* = −0.18
GHQ (total score)	*r* = −0.18	*r* = −0.17 *	*r* = −0.39 ***	*r* = −0.42 ***	*r* = −0.26

*r* = Spearman correlation coefficient, * p < 0.05, ** *p* < 0.01, *** *p* < 0.001.

**Table 12:** Correlations between KompOs—sense of power and other psychometric measures.

Measures of Depression, Mental Distress and Quality of Life	KompOs—Sense of Power and Other Psychometric Measures				
	Belarus	Poland	Greece	Turkey	Russia
Not pregnant women					
BDI	*r* = −0.47 ***	*r* = −0.26 **	*r* = −0.31 **	*r* = −0.29 ***	*r* = −0.44 ***
SWLS	*r* = 0.54 ***	*r* = 0.29 ***	*r* = 0.30 ***	*r* = 0.37 ***	*r* = 0.48 ***
GHQ (somatic symptoms)	*r* = −0.21 *	*r* = −0.08	*r* = −0.21 *	*r* = −0.23 **	*r* = −0.24 ***
GHQ (anxiety, insomnia)	*r* = −0.15	*r* = −0.10	*r* = −0.34 ***	*r* = −0.30 ***	*r* = −0.24 ***
GHQ (dysfunctions)	*r* = −0.31 ***	*r* = −0.35 ***	*r* = −0.29 ***	*r* = −0.31 ***	*r* = −0.32 ***
GHQ (depressive symptoms)	*r* = −0.29 ***	*r* = −0.19 *	*r* = −0.16	*r* = −0.27 ***	*r* = −0.26 ***
GHQ (total score)	*r* = −0.23 **	*r* = −0.15	*r* = −0.30 ***	*r* = −0.34 ***	*r* = −0.31 ***
Pregnant women					
BDI	*r* = −0.24 **	*r* = −0.19 *	*r* = −0.31 **	*r* = −0.51 ***	*r* = −0.14
SWLS	*r* = 0.34 ***	*r* = 0.28 ***	*r* = 0.48 ***	*r* = 0.56 ***	*r* = 0.15
GHQ (somatic symptoms)	*r* = 0.07	*r* = −0.17 *	*r* = −0.18	*r* = −0.44 ***	*r* = −0.06
GHQ (anxiety, insomnia)	*r* = −0.09	*r* = −0.21 **	*r* = −0.20 *	*r* = −0.37 ***	*r* = −0.21
GHQ (dysfunctions)	*r* = −0.09	*r* = −0.14	*r* = −0.19	*r* = −0.37 ***	*r* = 0.31
GHQ (depressive symptoms)	*r* = −0.08	*r* = −0.11	*r* = −0.34 ***	*r* = −0.34 ***	*r* = 0.14
GHQ (total score)	*r* = −0.03	*r* = −0.22 **	*r* = −0.23 *	*r* = −0.47 ***	*r* = 0.02
Postpartum women					
BDI	*r* = −0.31 **	*r* = −0.34 ***	*r* = −0.28 **	*r* = −0.46 ***	*r* = −0.08
EPDS	*r* = −0.44 ***	*r* = −0.43 ***	*r* = −0.49 ***	*r* = −0.37 ***	*r* = −0.06
SWLS	*r* = 0.28 *	*r* = 0.32 ***	*r* = 0.36 ***	*r* = 0.59 ***	*r* = 0.19
GHQ (somatic symptoms)	*r* = −0.12	*r* = −0.25 **	*r* = −0.15	*r* = −0.54 ***	*r* = 0.09
GHQ (anxiety, insomnia)	*r* = −0.16	*r* = −0.18 *	*r* = −0.22 *	*r* = −0.31 **	*r* = 0.02
GHQ (dysfunctions)	*r* = −0.14	*r* = −0.32 ***	*r* = −0.26 **	*r* = −0.42 ***	*r* = −0.04
GHQ (depressive symptoms)	*r* = 0.07	*r* = −0.29 ***	*r* = −0.44 ***	*r* = −0.51 ***	*r* = −0.03
GHQ (total score)	*r* = −0.18	*r* = −0.28 ***	*r* = −0.28 **	*r* = −0.49 ***	*r* = 0.02

*r* = Spearman correlation coefficient * *p* < 0.05, ** *p* < 0.01, *** *p* < 0.001.

**Table 13:** Correlations between KompOs and the sense of perseverance and other psychometric measures.

Measures of Depression, Mental Distress and Quality of Life	KompOs—Sense of Perseverance and Other Psychometric Measures				
	Belarus	Poland	Greece	Turkey	Russia
Not pregnant women					
BDI	*r* = −0.24 **	*r* = −0.19 *	*r* = 0.01	*r* = −0.27 ***	*r* = −0.22 ***
SWLS	*r* = 0.31 ***	*r* = 0.06	*r* = 0.06	*r* = 0.37 ***	*r* = 0.22 ***
GHQ (somatic symptoms)	*r* = 0.09	*r* = −0.09	*r* = 0.09	*r* = −0.27 ***	*r* = −0.12 *
GHQ (anxiety, insomnia)	*r* = 0.07	*r* = −0.04	*r* = −0.01	*r* = −0.24 **	*r* = −0.15 **
GHQ (dysfunctions)	*r* = −0.16	*r* = −0.19 *	*r* = −0.14	*r* = −0.22 **	*r* = −0.22 ***
GHQ (depressive symptoms)	*r* = −0.08	*r* = −0.24 **	*r* = 0.02	*r* = −0.21 *	*r* = −0.14 *
GHQ (total score)	*r* = 0.03	*r* = −0.11	*r* = 0.0 1	*r* = −0.29 ***	*r* = −0.19 ***
Pregnant women					
BDI	*r* = −0.11	*r* = 0.01	*r* = −0.22 *	*r* = −0.47 ***	*r* = −0.24
SWLS	*r* = 0.07	*r* = 0.04	*r* = 0.40 ***	*r* = 0.44 ***	*r* = 0.01
GHQ (somatic symptoms)	*r* = 0.03	*r* = −0.02	*r* = −0.20 *	*r* = −0.36 ***	*r* = 0.19
GHQ (anxiety, insomnia)	*r* = −0.02	*r* = −0.10	*r* = −0.19	*r* = −0.37 ***	*r* = 0.09
GHQ (dysfunctions)	*r* = −0.08	*r* = −0.03	*r* = −0.30 **	*r* = −0.25 *	*r* = −0.01
GHQ (depressive symptoms)	*r* = 0.10	*r* = −0.16	*r* = −0.29 **	*r* = −0.25 *	*r* = −0.17
GHQ (total score)	*r* = −0.04	*r* = −0.08	*r* = −0.25 **	*r* = −0.45 ***	*r* = 0.11
Postpartum women					
BDI	*r* = −0.17	*r* = −0.13	*r* = −0.17	*r* = −0.35 ***	*r* = 0.03
EPDS	*r* = −0.34 **	*r* = −0.23 **	*r* = −0.46 ***	*r* = −0.29 **	*r* = 0.08
SWLS	*r* = 0.28 *	*r* = 0.22 **	*r* = 0.26 **	*r* = 0.52 ***	*r* = 0.00
GHQ (somatic symptoms)	*r* = −0.13	*r* = −0.01	*r* = −0.20 *	*r* = −0.49 ***	*r* = 0.18
GHQ (anxiety, insomnia)	*r* = −0.15	*r* = 0.03	*r* = −0.21 *	*r* = −0.16	*r* = 0.16
GHQ (dysfunctions)	*r* = −0.06	*r* = −0.07	*r* = −0.37 ***	*r* = −0.34 ***	*r* = 0.01
GHQ (depressive symptoms)	*r* = −0.03	*r* = −0.25 **	*r* = −0.33 ***	*r* = −0.39 ***	*r* = −0.13
GHQ (total score)	*r* = −0.03	*r* = −0.03	*r* = −0.30 **	*r* = −0.39 ***	*r* = 0.18

*r* = Spearman correlation coefficient, * p < 0.05, ** *p* < 0.01, *** *p* < 0.001.

**Table 14:** Correlations between KompOs—general measure and other psychometric measures.

Measures of Depression, Mental Distress and Quality of Life	General Measure of Personal Competence and Other Psychometric Measures				
	Belarus	Poland	Greece	Turkey	Russia
Not pregnant women					
BDI	*r* = −0.43 ***	*r* = −0.27 **	*r* = −0.20	*r* = −0.33 ***	*r* = −0.40 ***
SWLS	*r* = 0.51 ***	*r* = 0.22 *	*r* = 0.24 **	*r* = 0.44 ***	*r* = 0.43 ***
GHQ (somatic symptoms)	*r* = −0.08	*r* = −0.10	*r* = −0.08	*r* = −0.30 ***	*r* = −0.21 ***
GHQ (anxiety, insomnia)	*r* = −0.06	*r* = −0.09	*r* = −0.22 *	*r* = −0.32 ***	*r* = −0.25 ***
GHQ (dysfunctions)	*r* = −0.29 ***	*r* = −0.33 ***	*r* = −0.28 **	*r* = −0.31 ***	*r* = −0.33 ***
GHQ (depressive symptoms)	*r* = −0.22 *	*r* = −0.26 **	*r* = −0.10	*r* = −0.29 ***	*r* = −0.23 ***
GHQ (total score)	*r* = −0.13	*r* = −0.16	*r* = −0.19 *	*r* = −0.38 ***	*r* = −0.29 ***
Pregnant women					
BDI	*r* = −0.20 *	*r* = −0.14	*r* = −0.28 **	*r* = −0.56 ***	*r* = −0.28
SWLS	*r* = 0.24 **	*r* = 0.20 *	*r* = 0.47 ***	*r* = 0.57 ***	*r* = 0.03
GHQ (somatic symptoms)	*r* = 0.05	*r* = −0.13	*r* = −0.20 *	*r* = −0.43 ***	*r* = 0.10
GHQ (anxiety, insomnia)	*r* = −0.08	*r* = −0.20 *	*r* = −0.21 *	*r* = −0.43 ***	*r* = −0.05
GHQ (dysfunctions)	*r* = −0.10	*r* = −0.11	*r* = −0.27 **	*r* = −0.35 ***	*r* = 0.11
GHQ (depressive symptoms)	*r* = 0.02	*r* = −0.20 *	*r* = −0.34 ***	*r* = −0.34 ***	*r* = −0.10
GHQ (total score)	*r* = −0.05	*r* = −0.20 *	*r* = −0.26 **	*r* = −0.52 ***	*r* = 0.06
Postpartum women					
BDI	*r* = −0.29 *	*r* = −0.28 ***	*r* = −0.26 **	*r* = −0.45 ***	*r* = −0.06
EPDS	*r* = −0.49 ***	*r* = −0.38 ***	*r* = −0.52 ***	*r* = −0.39 ***	*r* = −0.01
SWLS	*r* = 0.31 **	*r* = 0.33 ***	*r* = 0.37 ***	*r* = 0.62 ***	*r* = 0.13
GHQ (somatic symptoms)	*r* = −0.21	*r* = −0.17 *	*r* = −0.20 *	*r* = −0.56 ***	*r* = 0.10
GHQ (anxiety, insomnia)	*r* = −0.24 *	*r* = −0.09	*r* = −0.25 *	*r* = −0.25 **	*r* = 0.12
GHQ (dysfunctions)	*r* = −0.12	*r* = −0.26 **	*r* = −0.35 ***	*r* = −0.42 ***	*r* = −0.04
GHQ (depressive symptoms)	*r* = 0.04	*r* = −0.35 ***	*r* = −0.43 ***	*r* = −0.50 ***	*r* = −0.10
GHQ (total score)	*r* = −0.25 *	*r* = −0.20 *	*r* = −0.34 ***	*r* = −0.48 ***	*r* = 0.09

*r* = Spearman correlation coefficient, * *p* < 0.05, ** *p* < 0.01, *** *p* < 0.001.

## Data Availability

The data presented in this study are available on request from the corresponding author.

## Supplementary Materials

The following supporting information can be downloaded at: https://www.mdpi.com/article/10.3390/jcm13020533/s1, Table S1: Correlations between BDI and SWLS and age of women from different countries; Table S2: History of spontaneous and induced abortions; Table S3: Correlations between BDI and SWLS and having children among women, including pregnant women from each country.
