# Supplementary material for: Impact of Pregnancy on Self-Efficacy and Personal Competence in the Context of Risk of Depression, Mental Health Status, and Satisfaction with Life
Source: J Clin Med. 2024 Jan 17;13(2):533. doi: 10.3390/jcm13020533 (PMC10816068; doi:10.3390/jcm13020533)
Supplement: Supplementary file 1 [file jcm-13-00533-s001.zip › jcm-2760341-supplementary.pdf]

Table S1. Correlations between BDI and SWLS and age of women from different countries.

Country	Pregnant women	
	Age vs BDI	Age vs SWLS
Belarus	*r*= -0.02 (*p* = 0.8221)	*r*= -0.12 (*p* = 0.1432)
Poland	*r*= -0.07 (*p* = 0.3839)	*r*= 0.03 (*p* = 0.6893)
Greece	*r*= -0.03 (*p* = 0.7619)	*r*= -0.04 (*p* = 0.6572)
Turkey	*r*= -0.08 (*p* = 0.4137)	*r*= 0.03 (*p* = 0.7591)
Russia	*r*= -0.12 (*p* = 0.4323)	*r*= -0.06 (*p* = 0.6945)

Table S2. History of spontaneous and induced abortions.

	**Belarus N =250**		**Poland N =308**		**Greece N =230**		**Turkey N =210**		**Russia N =95**	
	**Pregnant**	**Postpartum**	**Pregnant**	**Postpartum**	**Pregnant**	**Postpartum**	**Pregnant**	**Postpartum**	**Pregnant**	**Postpartum**
	***N* = 147**	***N* = 103**	***N* = 173**	***N* = 163**	***N* = 114**	***N* = 106**	***N* = 103**	***N* = 107**	***N* = 47**	***N* = 48**
**Spontaneous abortions**										
None	125	87	134	129	87	75	121	107	38	40
yes	22	16	37	34	27	31	29	0	9	8
1 miscarriage	19	14	30	26	25	26	17	0	9	8
2 miscarriages	1	2	4	4	2	5	11	0	0	0
3 miscarriages	2	0	3	4	0	0	1	0	0	0
Total	147	103	171	163	114	106	103	107	47	48
**Induced abortions**										
	**Belarus *N* = 250**		**Poland *N* = 308**		**Greece *N* = 230**		**Turkey *N* = 210**		**Russia *N* = 95**	
	Pregnant	Postpartum	Pregnant	Postpartum	Pregnant	Postpartum	Pregnant	Postpartum	Pregnant	Postpartum
	N =147	*N* =103	*N* =173	*N* =163	*N* =114	*N* =106	*N* =103	*N* =107	*N* =47	*N* =48
None	130	91	171	163	104	95	94	107	44	46
yes	17	12	0	0	10	11	9	0	3	2
1 miscarriage	12	10	0	0	7	7	9	0	2	2
2 miscarriages	3	1	0	0	3	2	0	0	1	0
3 miscarriages	2	0	0	0	0	2	0	0	0	0
5 miscarriages	0	1	0	0	0	0	0	0	0	0
Total	147	103	171	163	114	106	103	107	47	48

Table S3. Correlations between BDI and SWLS and having children among women, including pregnant women from each country.

Having children															
	Belarus			Poland			Greece			Turkey			Russia		
no	48.3%			43.4%			16.7%			58.3%			61.7%		
yes	51.7%			56.6%			83.3%			41.7%			38.3%		
Average number of children	1.5			1.7			2.0			1.6			1.2		
**Beck Depression Inventory vs Having children**															
	**Belarus**			**Poland**			**Greece**			**Turkey**			**Russia**		
	$\bar{x}$	Me	IQR	$\bar{x}$	Me	IQR	$\bar{x}$	Me	IQR	$\bar{x}$	Me	IQR	$\bar{x}$	Me	IQR
yes	6.4	5	7.0	6.0	4	7.0	8.2	7	8.0	9.2	9	8.0	7.1	5	6.0
no	6.0	5	5.0	5.8	5	7.0	8.9	9	5.0	7.9	8	5.0	7.9	6.5	7.0
p	0.9074			0.6603			0.3549			0.1830			0.5701		
**SWLS vs Having children**															
	**Belarus**			**Poland**			**Greece**			**Turkey**			**Russia**		
	$\bar{x}$	Me	IQR	$\bar{x}$	Me	IQR	$\bar{x}$	Me	IQR	$\bar{x}$	Me	IQR	$\bar{x}$	Me	IQR
yes	26.1	26	7.0	25.1	25.5	7.0	25.2	26	9.0	27.7	28	5.0	25.4	26	9.0
no	26.8	27	6.0	25.3	25	7.0	24.4	27	12.0	29.2	29	4.0	26.8	27	5.0
p	0.3390			0.9987			0.7600			0.0623			0.3888		

Notes $\bar{x}$ – Mean , Me – Median, IQR- interquartile range
